# In silico prediction of potential indigenous microbial biomarkers in *Penaeus vannamei* identified through meta-analysis and genome-scale metabolic modelling

**DOI:** 10.1186/s40793-022-00458-6

**Published:** 2023-01-11

**Authors:** Neelakantan Thulasi Devika, Vinaya Kumar Katneni, Ashok Kumar Jangam, Panjan Nathamuni Suganya, Mudagandur Shashi Shekhar, Karingalakkandy Poochirian Jithendran

**Affiliations:** 1 Nutrition Genetics and Biotechnology Division, Indian Council of Agricultural Research - Central Institute of Brackishwater Aquaculture, Chennai, India; 2 Aquatic Animal Health and Environment Division, Indian Council of Agricultural Research - Central Institute of Brackishwater Aquaculture, Chennai, India

**Keywords:** *Penaeus vannamei*, Meta-analysis, 16S amplicon sequence analysis, Metagenomics, Genome-scale metabolic modelling, Flux balance analysis, Flux variability analysis, Microbial biomarker

## Abstract

**Background:**

Understanding the microbiome is crucial as it contributes to the metabolic health of the host and, upon dysbiosis, may influence disease development. With the recent surge in high-throughput sequencing technology, the availability of microbial genomic data has increased dramatically. Amplicon sequence-based analyses majorly profile microbial abundance and determine taxonomic markers. Furthermore, the availability of genome sequences for various microbial organisms has prompted the integration of genome-scale metabolic modelling that provides insights into the metabolic interactions influencing host health. However, the analysis from a single study may not be consistent, necessitating a meta-analysis.

**Results:**

We conducted a meta-analysis and integrated with constraint-based metabolic modelling approach, focusing on the microbiome of pacific white shrimp *Penaeus vannamei*, an extensively cultured marine candidate species. Meta-analysis revealed that *Acinetobacter* and *Alteromonas* are significant indicators of "health" and "disease" specific taxonomic biomarkers, respectively. Further, we enumerated metabolic interactions among the taxonomic biomarkers by applying a constraint-based approach to the community metabolic models (4416 pairs). Under different nutrient environments, a constraint-based flux simulation identified five beneficial species: *Acinetobacter* spWCHA55, *Acinetobacter tandoii* SE63, *Bifidobacterium pseudolongum* 49 D6, *Brevundimonas pondensis* LVF1, and *Lutibacter profundi* LP1 mediating parasitic interactions majorly under sucrose environment in the pairwise community. The study also reports the healthy biomarkers that can co-exist and have functionally dependent relationships to maintain a healthy state in the host.

**Conclusions:**

Toward this, we collected and re-analysed the amplicon sequence data of *P. vannamei* (encompassing 117 healthy and 142 disease datasets). By capturing the taxonomic biomarkers and modelling the metabolic interaction between them, our study provides a valuable resource, a first-of-its-kind analysis in aquaculture scenario toward a sustainable shrimp farming.

**Supplementary Information:**

The online version contains supplementary material available at 10.1186/s40793-022-00458-6.

## Introduction

With the advent of high-throughput sequencing, metagenome datasets have become increasingly accessible. The culture-independent metagenomic approach has facilitated extensive analysis of microbiome data for preventive and diagnostic purposes. Even in the aquaculture sector, microbial consortiums are crucial for improving sustainability and productivity of aquatic organisms [5, 43]. Several factors threaten the sustainability and yield of aquaculture species, one of which relates to the infection caused by pathogenic microorganisms [29]. Therefore, understanding the essential and significant microbial consortia would simplify the prediction of disease incidences. In aquatic animals, amplicon sequence based approach has been used to identify microbiomes, their composition, and functions [4, 30, 33, 54].

The current study focuses on *Penaeus vannamei*, one of the world's most widely cultured and traded marine candidate species. *P. vannamei* farming has increased rapidly to meet the growing edible high-quality protein demand. Despite this, *P. vannamei* farming is constantly hampered by abiotic and biotic factors [58]. One of the challenges faced by *P. vannamei* farming relates to emerging diseases caused by bacteria, viruses, and fungi affecting the sustainability of shrimp [1, 31, 32]. Antibiotics are used to prevent the growth of microbial infections, but the spread of microbial antibiotic resistance poses a hazard to human health [5]. Alternatively, microbes also promote the host's growth by acting as a probiotic to circumvent antimicrobials [50]. Thus, studying the potential, resident, and beneficial microbes is crucial for improving host health. To date, several amplicon sequence-based studies on *P. vannamei* have characterized the microbial communities in different habitats [6], developmental stages [6], disease conditions [59], and diet [47].

It is important to note that the conclusions drawn from a single study may not accurately reflect the microbial communities within the host [15]. Therefore, we performed a meta-analysis to gain a more robust and consistent understanding of health vs. disease states in *P. vannamei*. Previous microbiome-based meta-analyses targeted different ontogenic stages of the healthy and disease shrimp predicting taxonomic markers [56]. Another study revealed the role of different biological factors like environment and different life stages in shaping the microbiota [9]. Furthermore, these studies might not encapsulate the interrelationships between microbes for maintaining a stable microbial community. Therefore, it is necessary to profile and explore the microbial interactions that can mediate beneficial host functions. Constraint-based metabolic modelling with genome-scale metabolic models (GSM) has been a widely used approach to study microbial interactions between species in a community [13, 28, 49]. Moreover, reconstructing genome-scale metabolic networks from amplicon sequence data has also been employed to infer resource competition and metabolic cooperation potential in microbial consortia [36, 60]. Hence, employing, flux balance analysis on the genome-scale metabolic models allow assessment of nutritional requirements, interactions, and metabolic exchanges in a microbial consortia under diverse nutrient environments [3, 60]. Moreover, a microbial community's ability to coexist in different habitats depends on the cross-feeding of metabolites [57]. Natural metabolic products such as short-chain fatty acids have been used to control pathogenic bacterial growth [39, 48]. Likewise, using short-chain fatty acids in aquaculture also provides a growth advantage to *Penaeus vannamei* by suppressing the growth of pathogenic species [21].

Most microbiome-based studies have identified biomarkers associated with healthy and disease shrimp; however, this study is the first to integrate 16S amplicon sequence data and genome-scale metabolic modelling in aquaculture scenario to capture the metabolic interaction between the taxonomic biomarker species. The goal is to identify the most efficient taxonomic biomarkers that, in turn, should also limit the growth of pathogenic biomarker species. In summary, applying multi-pronged approach, we systematically characterized the natural indigenous species eventually coexist in the host or need to be artificially introduced to the host towards a sustainable health management.

## Methods

### Public data collection

The amplicon sequence data used for this meta-analysis were retrieved from the NCBI SRA database (Downloaded as on September, 2021). The studies collected were related to *P. vannamei* species associated with a disease, considering only the host tissue or intestine samples. Initially, the search yielded 13 studies with 838 datasets [Additional file 1: Table S1]. However, several studies were not considered due to lack of habitat information. The disease dataset included in this meta-analysis are infected with White feces disease events (WFD), White Spot Syndrome Virus (WSSV) and Acute Hepatopancreatic Necrotic Disease (AHPND). The available studies differed in the sub-regions of the 16S rRNA gene, such as V3-V4, V4, V1-V2, V2 mix, and V3 mix sequenced with different sequencing platforms. Due to the limited number of studies with proper metadata, a uniform sequencing technology or a common hypervariable region could not be implemented. Finally, six studies totaling 259 datasets were considered [Additional file 1: Table S2].

### Microbiome analysis

Initially, each study was processed separately, with single-end and paired-end sequences analyzed using the Quantitative Insights Into Microbial Ecology (QIIME2) pipeline [17]. The read quality assessment was conducted with the DADA2 plugin to weed out low-quality forward and reverse reads that did not meet the quality threshold of 20. For DADA2, the trimming and truncation parameters unique to each study were provided. The datasets were removed when the read quality dropped below the threshold. The filtered reads from each study were aligned and classified using the SILVA database [55]. Finally, Amplicon Sequence Variants (ASV), a higher resolution version of the Operational Taxonomic Unit (OTU) was generated. Next, we combined the dataset wherein individual studies were merged. We filtered out datasets that fall below 2000 reads from the combined dataset and removed low abundance features (removed features that appeared in less than ten datasets). In the end, 241 datasets from five studies were analysed. Next, the representative sequences obtained from the combined pre-processed data were assigned taxonomy using the pre-trained classifier SILVA (Silva release 138; 99% OTUs full-length sequences). Also, taxon archaea and eukaryota were not included as part of our analysis. In addition, chloroplast, mitochondria, and unassigned genus were removed from the feature table.

### Alpha- and beta-diversity

The within-dataset difference was measured using alpha diversity in individual and combined dataset. For individual studies and combined dataset Shannon, Chao1, Observed Features, and Simpson were estimated to determine the community richness and evenness between the datasets. With the help of the vegan package in R (ggplot), beta-diversity was quantified using Bray–Curtis dissimilarity and visualized through nonmetric multidimensional scaling (NMDS). The beta-diversity was also measured with unweighted unifrac distance metrics. The statistical difference between the healthy and disease states was computed with the Kruskal–Wallis for alpha diversity metrics and Permutational Multivariate Analysis of Variance (PERMANOVA) for both the beta-diversity metrics. A *p-*value less than 0.05 was considered as significant.

### Identification of biomarker

A taxonomic biomarker that can differentiate between a healthy and a disease state was found using the linear discriminant analysis Effect Size (LEfSe) method [46]. ASV tables derived from combined datasets were filtered for unassigned/uncultured genera before subjecting to LEfSe analysis. An effect size (LDA score) of > 2.0 with statistically significant *p*-value (< 0.05) was used for biomarker identification. A taxon was more accurately distinguished between its respective healthy and disease states when it has a large effect size and is statistically significant in a set of datasets.

### Computing co-occurrence

The relative abundance data corresponding to healthy and disease biomarker identified with LEfSe were subjected to **Hi**gher-**Or**der **Co**-occurrence (HiOrCo) patterns in microbial samples for computing the highly co-occurring species [36]. The algorithm begins by considering the pairs of species that co-occur in samples and proceeds to a group of larger sizes and generate to a default of 100 communities in each size. The algorithm evaluates such that the species co-occur in at least twice in 10 datasets and should pass the FDR-correction test.

### Mapping ASV to prokaryotic database with complete genome

The 16 s rRNA sequences corresponding to the healthy/disease biomarkers were retrieved from the combined dataset. All the retrieved sequences were mapped to the complete bacterial genomes downloaded from NCBI RefSeq (23,764 complete genomes as on November 11, 2021). We carried out standalone BLASTP with an e-value cut off 10^–6^ and with a percent identity and query coverage of 97%. Species with hits matching the above criteria were selected for the downstream analysis such as for building metabolic models.

### Reconstruction of genome-scale metabolic model

The protein sequence that mapped to their closed reference genome were retrieved based on percent identity and query coverage [Additional file 1: Table S3]. A genome scale metabolic model was built separately for each of the species using CarveMe v1.2.2 [35]. Using top-down approach CarveMe constructs organism specific models after removal of reactions and metabolites that are absent in the target organism. A total of 123 individual models were reconstructed gap-filled, grown in M9 minimal media with glucose as the carbon source, assigned with biomass components specific to Gram-positive and Gram-negative bacteria [Additional file 1: Table S4]. The individual species assigned as disease biomarker were then merged pairwise with all possible pairs with the species assigned as healthy biomarker resulting in 4416 pairs.

### Simulation of GSM using flux balance and flux variability analysis

CobraPy [16], a constraint-based modelling package in python with *cplex* solver for solving the optimization problems was used for performing simulations. The in silico growth prediction of individual and pairwise models were performed with highly reliable flux balance analysis (FBA). FBA is a constraint-based modelling method that estimates the fluxes of reactions in a metabolic network to capture the metabolic capabilities of an organism [40]. FBA solves linear system of equations derived from the stoichiometric matrix $S_{m \times r}$, expressed mathematically as follows:

$$\begin{aligned} & Objective: \\ & \begin{array}{*{20}l} {{\text{Max}}\,\nu_{bio} } \hfill \\ {s.t\quad S.\nu = 0} \hfill \\ {\quad Lj < \nu j < Uj} \hfill \\ \end{array} \\ \end{aligned}$$

where *m* is the number of metabolites and *r* is the number of reactions, *v* represents the flux through all reactions, *Lj* and *Uj* are the lower and upper bound flux of each reaction *j*. For the in silico growth prediction, 17 different carbon sources [Additional file 1: Table S5] were used and allowed the uptake of single carbon source at a time with maximization of biomass as the objective function. The in silico growth simulation on different nutrient environments were performed by setting the lower bound of each of the carbon sources to -10 mmol/gDw/h and setting the lower bound of other carbon sources to 0. The lower bounds of amino acid exchange reactions and other essential components was set as -1 mmol/gDW/h [Additional file 1: Table S4]. The in silico growth rate of single and paired species are compared and observed for a 10 percent increase or decrease in growth of healthy/disease species in the presence of another [24].

Flux variability analysis (FVA) was used to predict the acetate production/consumption in the community under diverse nutrient environments. FVA computes the maximum and minimum flux range through each reaction with biomass reaction constrained to the maximum growth rate achieved [38].

$$\begin{array}{*{20}l} {Maximize,Minimize} \hfill & {vj} \hfill & {} \hfill \\ {} \hfill & {S.t.} \hfill & {S.v = 0} \hfill \\ {} \hfill & {} \hfill & {v_{j}^{min} \le v \ge v_{j}^{\max } } \hfill \\ \end{array}$$

where *v* represents the maximum and minimum flux through each reaction *j.* Since acetate is considered as an indicator which suppresses the growth of pathogenic species, we conducted FVA on acetate reaction. In a pairwise community, acetate was considered secreted by an organism, if the flux of acetate exchange reaction was positive, and consumed by the organism, if the flux of acetate exchange reaction was negative.

## Results

**Fig. 1:**
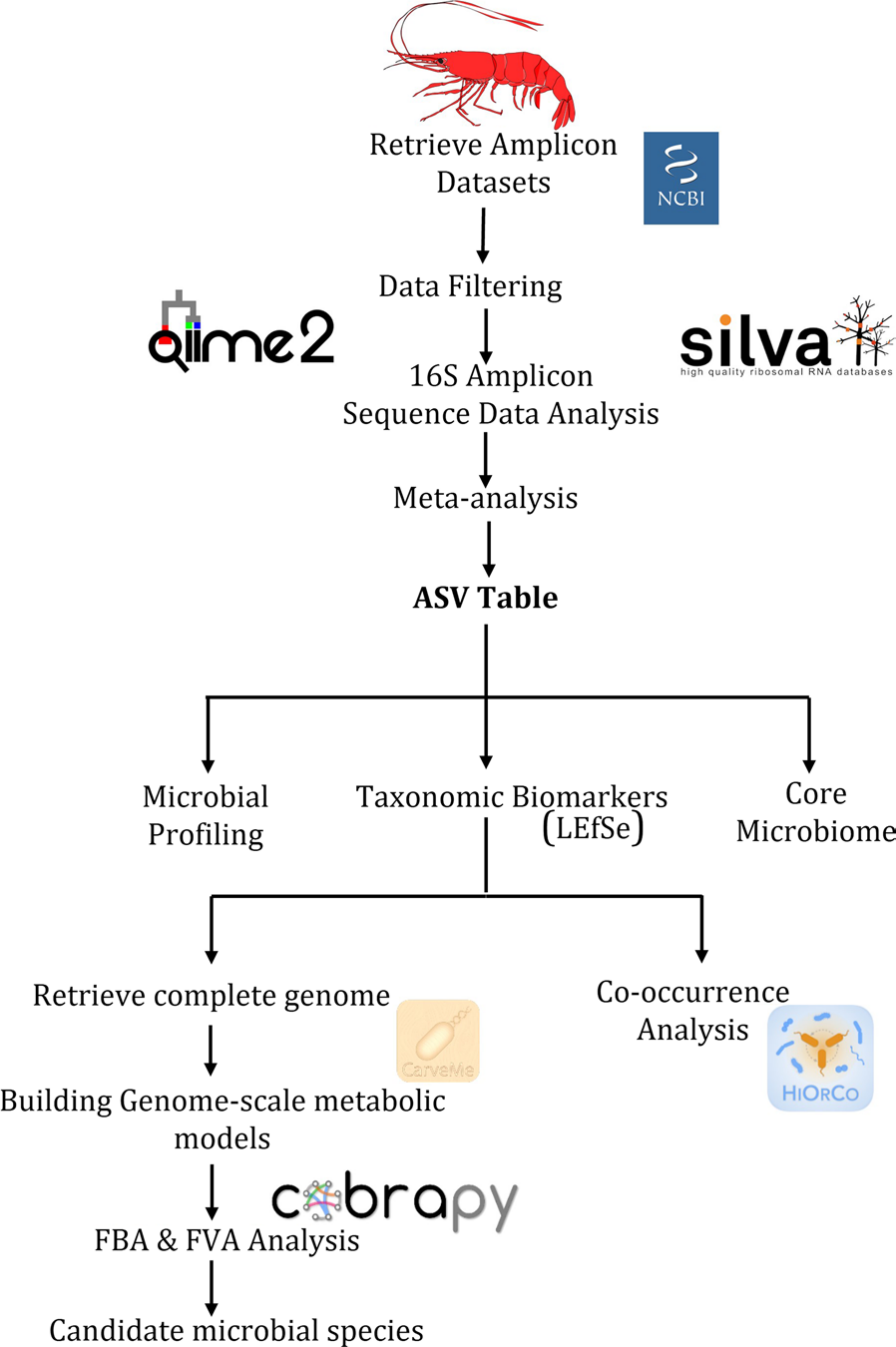
Outline of the study integrating meta-analysis and genome-scale metabolic modelling for identifying potential microbial species

The six studies included data spanning 259 datasets (117 Healthy & 142 Disease). The inclusion criteria for this meta-analysis were amplicon sequence-based studies on *P. vannamei* in healthy and disease states. For identifying a potential taxonomic biomarker, a single study may not be sufficient; hence we performed a meta-analysis that could serve as a representative and biologically meaningful biomarker. Additionally, we conducted constraint-based metabolic modelling approach to capture the metabolic capabilities of taxonomic biomarkers under different nutritional environments and inferred the interspecies interactions. Figure 1 depicts the workflow that outlines the key steps followed in this study.

### Diversity analysis for individual datasets

The data from each study were systematically processed and reanalyzed using the QIIME2 pipeline. Diversity measures, such as alpha and beta indices, were calculated based on taxonomic profiles obtained from each study. Based on the Shannon index [Additional file 2: Figure S1], healthy microbial datasets exhibited significantly higher richness and evenness (Kruskal–Wallis test, *p* < 0.05, four out of six studies) than disease microbial datasets [Additional file 1: Table S6]. Similarly, the beta diversity index unweighted unifrac distance tested with PERMANOVA revealed a significant difference (*p* < 0.05, four out of six studies) between the healthy and disease datasets [Additional file 1: Table S6]. We also assessed beta diversity metrics based on Bray–Curtis distance and visualized with NMDS plot [Additional file 2: Figure S2]. Briefly, four out of six studies demonstrated typical patterns of richness and diversity among the healthy and disease dataset. The phylum-level taxonomic profile exhibited a high abundance of *Proteobacteria* in five studies, while *Firmicutes* was abundant in one study.

### Diversity analysis for combined datasets

The datasets from six individual studies were combined and datasets with reads count below 2000 was removed, resulting in 241 datasets (one study was excluded due to low read counts). We computed alpha and beta diversity measures to investigate similarities within and between datasets. The alpha diversity metrics such as Shannon, Simpson, Chao1, ACE, and observed features computed for the combined dataset were presented in Additional file 1: Table S7. Shannon and Simpson index values revealed no significant difference (Kruskal–Wallis test, *p* > 0.05) between the healthy and disease states. With beta diversity metrics namely unweighted unifrac distance and Bray–Curtis distance, consistent results were observed, revealing a significant difference (PERMANOVA test, *p* < 0.05) between healthy and disease states. The Bray–Curtis distance which considered both species presence/absence and abundance was visualized through an NMDS plot [Additional file 2: Figure S3].

### Microbial abundance at phylum and genus level in healthy and disease states

**Fig. 2:**
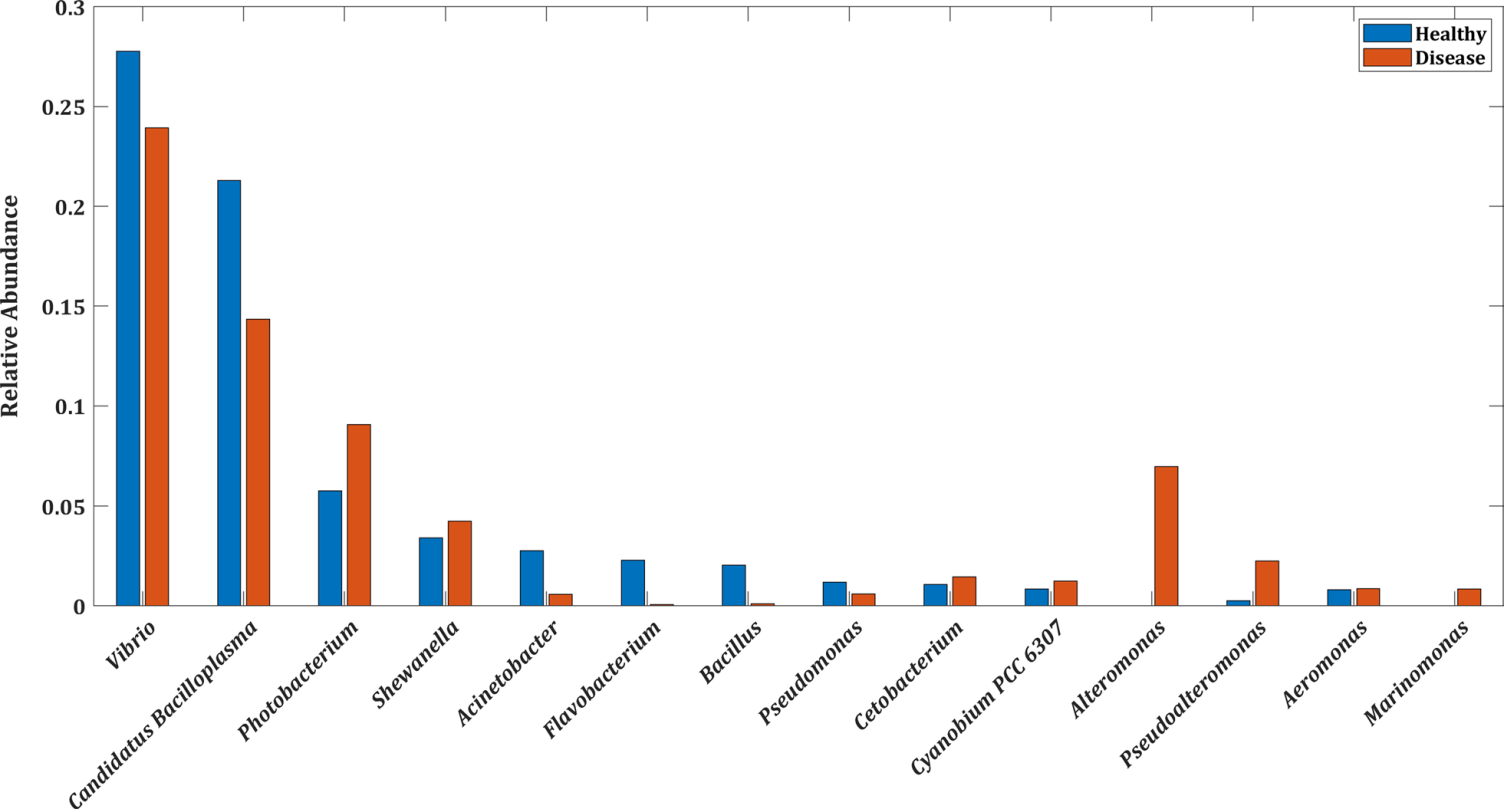
Boxplot depicting the mean relative abundance of top 10 genus-level taxa in healthy and disease states

We examined the top five dominant phylum and genera by computing the mean relative abundance across healthy and disease datasets. A phylum or genera with a mean abundance of ≥ 0.01 was considered abundant. At the phylum level, *Proteobacteria* (62% in healthy vs. 72% in disease state) and *Firmicutes* (30% in healthy vs. 20% in disease state) are the major representatives followed by *Bacteroidota*, *Actinobacteriota*, and *Cyanobacteria* [Additional file 2: Figure S4] in both the states based on the mean relative abundance. Four genera namely, *Vibrio, Candidatus Bacilloplasma*, *Photobacterium*, and *Shewanella* dominated both healthy and disease states in descending order of magnitude. However, the mean relative abundance of these genera was marginally different in both states, as shown in Fig. 2. Interestingly, the genus *Alteromonas* appeared only in the disease state, and the genus *Acinetobacter*, on the other hand, was more abundant in the healthy state. The disease state, however, showed a lower abundance of *Acinetobacter*.

### Identifying the core microbiome

We then analyzed the core microbiome (refers to a taxon that must appear across dataset) to identify the key genera associated with healthy and disease states. The genus *Vibrio* of *Proteobacteria* phylum was universally prevalent across healthy and disease state. However, owing to heterogeneous nature of the dataset, our study considered the microbes that are present in at least 50% of the dataset while looking across the healthy and disease state. Among the phyla, *Firmicutes*, *Proteobacteria*, *Actinobacteriota*, *Bacteroidota*, *Cyanobacteria*, and *Planctomycetota* were highly abundant in both the states, making them core phyla in *P. vannamei* regardless of the health state of the host. The core microbiome analysis at the genus level identified 24 and 19 genera at a prevalence of 50% in the healthy and disease state, respectively. Among these genera, 17 were common in both the states and comprise the core genera associated with *P. vannamei* irrespective of the health state of the host. Out of the 43 core genera present in both states, seven (*Gemmobacter*, *Chryseomicrobium*, *Stenotrophomonas*, *LD29*, *Sva0081_sediment_group*, *PLTA13*, *SZB30*) and two (*PeM15*, *Pseudoalteromonas*) genera were prevalent exclusively in the healthy and disease state, respectively [Additional file 2: Figure S5]. Interestingly, the genus *Acinetobacter* was present at a sample prevalence of 80% in healthy state, while 65% in disease state.

### Microbial biomarker detection in healthy and disease states

**Fig. 3:**
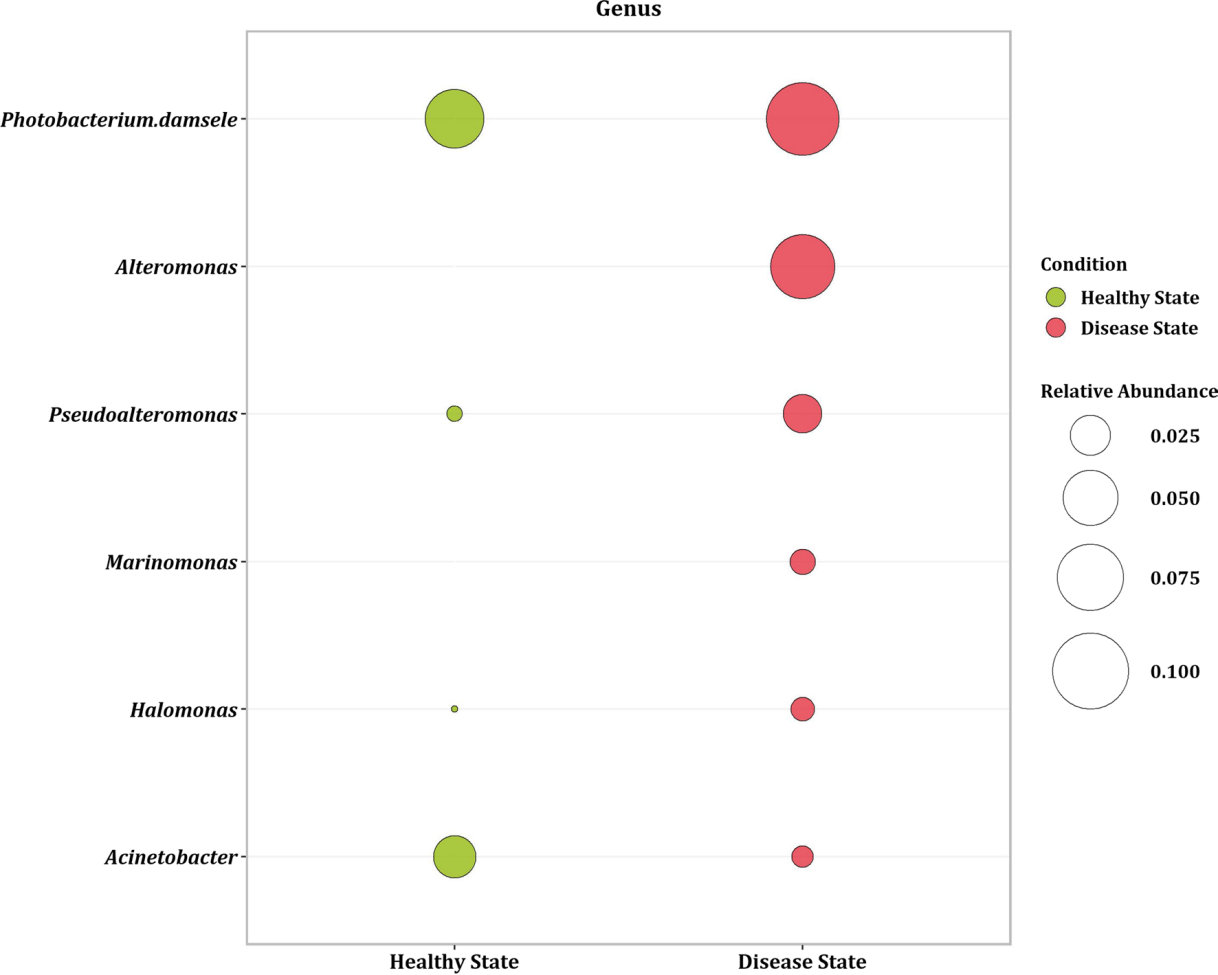
Plot depicting the mean relative abundance of dominant biomarker at genus level in healthy and disease state. Size of the circle represent the mean relative abundance

LEfSe analysis was carried out on the combined dataset to estimate whether there was a significant difference in the relative abundance between the healthy/disease state. LEfSe identified 32 beneficial and 73 disease genera [Additional file 1: Table S8] as prospective biomarkers with an effect size greater than two and a *p*-value < 0.05 [Additional file 2: Figure S6]. The top five healthy biomarker belonged to the phylum *Firmicutes*, and *Proteobacterium*, including genus, *Candidatus Bacilloplasma, Acinetobacter*, *Exiguobacterium*, *Lactobacillus*, and *Shimia*. On the other hand, the top five disease biomarker belonged to phylum *Proteobacteria* including genera *Alteromonas*, *Photobacterium*, *Pseudoalteromonas*, *Halomonas*, and *Marinomonas*. The mean relative abundance corresponding to the dominant taxonomic biomarkers in the healthy and disease state is depicted in Fig. 3. Further, it was interesting to note that most of the disease biomarkers identified belonged to the phylum *Proteobacteria*.

### Microbial biomarker co-occurrence

The significantly co-occurring microbial biomarkers essential in maintaining host health were determined using HiOrCo. The HiOrCo algorithm computed the co-occurring communities found together in at least ten datasets more often than expected by chance in healthy [Additional file 1: Table S9a] and disease state [Additional file 1: Table S9b]**.** Overall, the healthy biomarkers were co-occurring to a consortia size of 15 (genera up to 15). The genera, namely *PLTA13* (identified as *Thiohalobacter thiocyanaticus* strain Hrh1 based on BLAST similarity analysis—92%), *Chryseomicrobium, Lactobacillus, Bifidobacterium, Phormidesmis, Rubrobacter, Lutibacter, Exiguobacterium, Legionella,* and *Acinetobacter* were co-occurring in a consortium of maximum size and can be considered as the representative consortium indicative of health. Whereas, the disease-specific biomarkers co-occur in up to 26 genera. The genera, namely *Marinomonas*, *Pseudoalteromonas Candidatus*, *Aestuariibacter*, *Alcanivorax, Seonamhaeicola, PeM15* (identified as *Geodermatophilus ruber strain DSM 45317* based on BLAST similarity analysis—94%)*,* and *CL500-3* (identified as *Mucisphaera calidilacus* strain Pan265 based on BLAST similarity analysis -88%) were some dominant co-occurring disease biomarker communities, which should be steered effectively to control the pathogenic state of the host.

### Pairwise interactions between the taxonomic biomarkers

The 16 s rRNA sequences corresponding to the healthy/disease biomarkers identified with LeFSe were mapped to their closest reference genomes (prokaryotic database of bacterial species with a complete genome availability was created). Mapping with BLASTP based on percent identity and query coverage (97% similarity) retrieved 64 healthy (corresponding to 26 different genera) and 69 disease species (corresponding to 19 different genera).

A genome-scale metabolic model was built with CarveMe for the retrieved species and subjected to in silico phenotypic growth predictions on the single and pairwise (4416 pairs) species in a minimal media supplemented with 17 different carbon sources. Each of the paired models was analysed for an increase in growth rate for the healthy species (10% increase in in silico growth compared to the single) with a concomitant growth limit for the disease species (10% decrease in silico growth rate compared to single) in each of the environments.

**Fig. 4:**
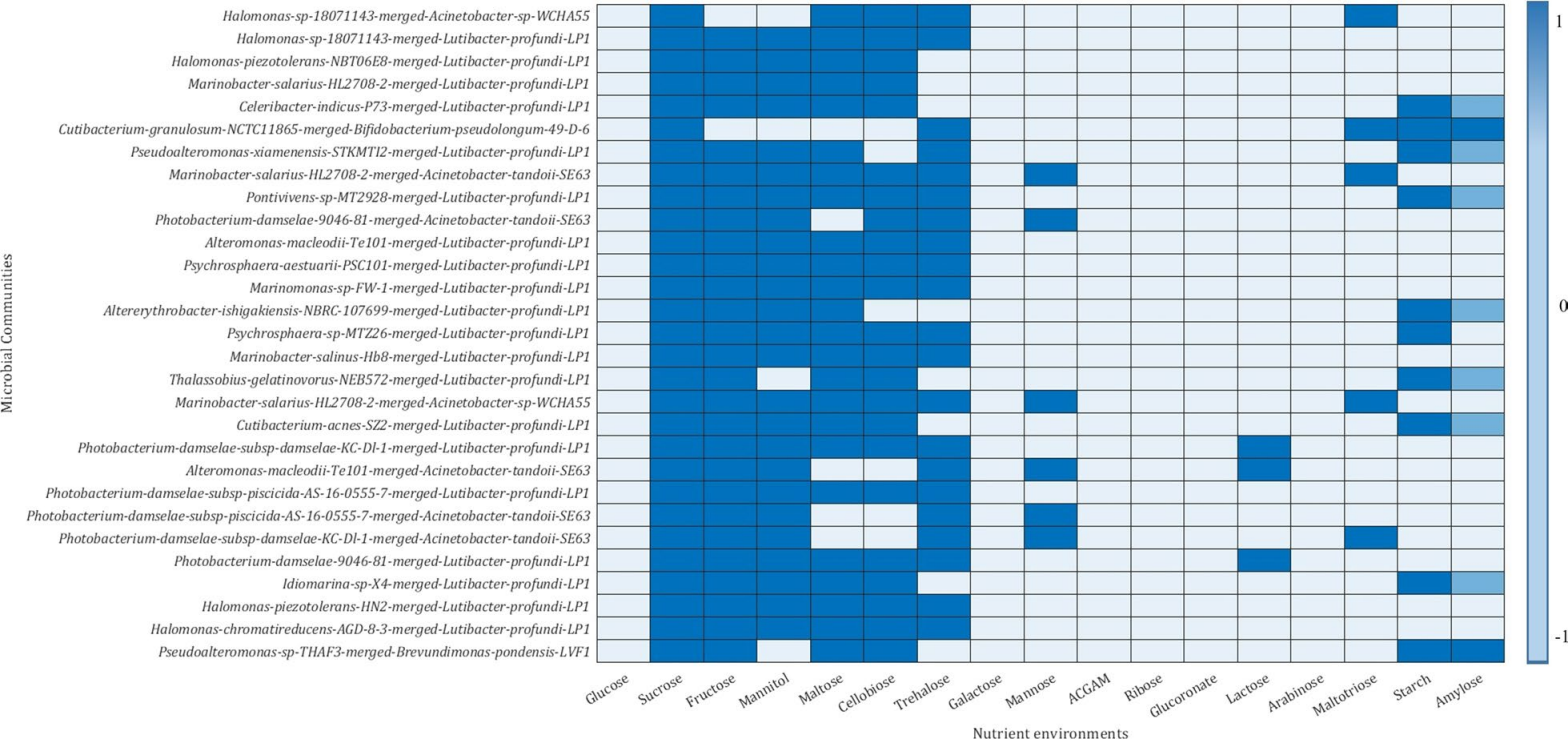
Heatmap depicting the growth suppression of disease species in a pairwise community. The dark blue represents the environment in which healthy species growth rate is 10% increase and growth rate of disease species is 10% decreased in pairwise community model

The healthy 69 species exhibited simulated growth capability in all the nutrient environments used in this study. On the other hand, the disease species exhibited less growth preference in sucrose, mannitol, and fructose environment. Further, out of the 4416 paired communities generated, only 794 pairs showed significant growth change and growth limitation on the healthy and disease species, respectively, in at least one of the nutrient environments. A total of 47 healthy species comprising 12 different genera form part of this 794 pairwise communities that limited the disease counterpart. These 47 healthy species limited the growth of 63 out of the 64 disease species except the strain *Synechococcus* CBW 1004. These growth limitations were majorly (711 paired communities) observed under sucrose environment, followed by trehalose (103 pairwise communities) and maltose (75 pairwise communities). Moreover, 29 out of the 794 pairwise microbial biomarker communities limited the growth of disease species in at least five of the nutrient environments [Fig. 4]. The five healthy species, namely *Acinetobacter* sp WCHA55, *Acinetobacter tandoii* SE63, *Bifidobacterium pseudolongum* 49 D 6, *Brevundimonas pondensis* LVF1, and *Lutibacter profundi* LP1 form part of 29 communities which limited the growth of 22 disease species in at least five of the nutrient environments. Among the five strains, *Lutibacter profundi* LP1 limited the growth of maximum number of disease species (20 different species). Altogether, these results short-listed four genera indigenous in *P. vannamei*, limiting the growth of disease species that naturally resides on the host.

### Cross-feeding of acetate in the pairwise communities

Flux variability analysis performed on the pairwise communities indicated that the production/consumption of acetate depends on the species with which it was paired and the nutrient environments.

**Fig. 5:**
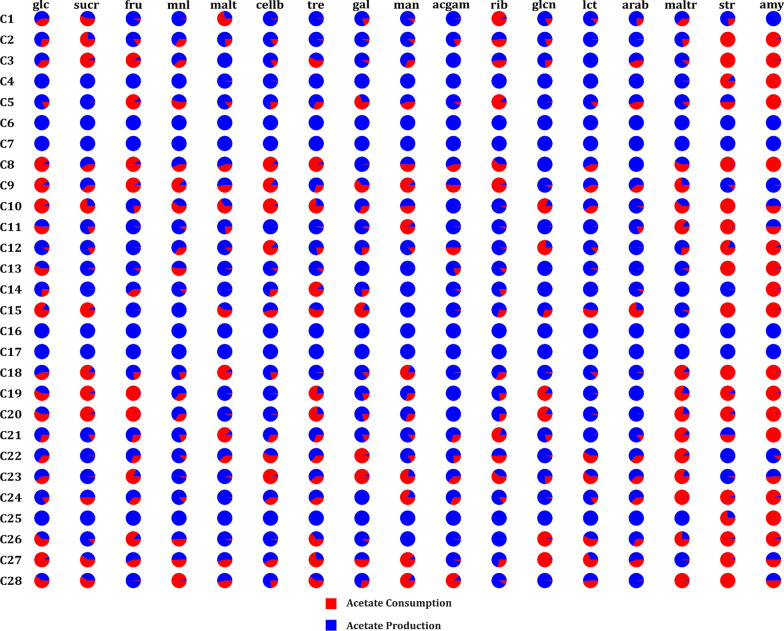
Pie chart depicting the production (denoted in blue) and consumption (denoted in red) of acetate under different nutrient environments in the shortlisted microbial communities. (Name corresponding to the pairwise microbial communities are provided in Additional file 1: Table S10)

The single and paired healthy species, namely, *Acinetobacter* sp WCHA55, *Acinetobacter tandoii* SE63, *Brevundimonas pondensis* LVF1, and *Lutibacter profundi* LP1 produced acetate under all nutrient environments. In the case of disease species, *Marinobacter salarius* HL27082 lost the ability to produce acetate upon paired with *Acinetobacter tandoii* SE63 and with *Acinetobacter* sp WCHA55 under all environments. While upon pairing with *Lutibacter profundi* LP1, *Marinobacter salaries* HL2708_2 could produce acetate in all the nutrient environments. Among the nutrient environments, glucose, fructose, and mannitol were associated with the production of acetate (as the acetate exchange reaction carry a positive flux) by the healthy species (Fig. 5). On the other hand, the disease species were consumers of acetate (as the acetate exchange reaction carry a negative flux) under these environments. Conversely, disease species produced acetate under maltotriose, amylose, and starch environments (as the acetate exchange reaction carry a positive flux).

## Discussion

Naturally existing microbial communities perform several vital tasks, such as degradation of organic matter, recycling the nutrients, and controlling the development of microbial infections [5]. Hence, a fundamental understanding of the health and disease-associated microbial communities for maintaining host health is required. Numerous 16S amplicon sequence-based studies have attempted to unravel the role of microbes, but findings drawn from one study may not decipher the full spectrum of microbes responsible for health/disease states. Hence, analyzing multiple studies in a meta-analysis has the potential to make inferences about common features associated with host health across different habitats. Although, several works were reported on human health perspective, very few studies are conducted on aquatic species, but did not extend to understand the metabolic interactions among them [9, 56]). Identifying taxonomic biomarkers in aquaculture is not new,however, most research focuses on single-study-based taxonomic biomarkers [27, 44], implying the need for a much comprehensive study. In this context, we performed a meta-analysis to identify the core taxonomic biomarkers that drive differences in the health/disease states in *P. vannamei* and determine the inter-species interactions exhibited by the microbial biomarkers under different nutrient environments. As an augmentation to the sustainable disease management, this study aims to identify beneficial indigenous species in *P. vannamei* essential for maintaining a good healthy state and controlling the growth of pathogenic species.

The study identified *Acinetobacter* as one of the prevalent and dominant genera in the healthy state. *Acinetobacter* was previously detected and reported in healthy shrimp to aid in suppressing harmful bacteria [18]. Furthermore, the leading genera revealed in this study's healthy state, namely *Acinetobacter* and *Candidatus Bacilloplasma,* were reported to act as a central hub connecting the significant bacterial population in the network of healthy shrimp hepatopancreas [51]. Further, *Exiguobacterium* genera in the healthy state have improved growth and survival in *P. vannamei* and provided probiotic advantages [7, 11]. *Lactobacillus* members identified in this study are known probiotics and have been extensively studied for their abilities to maintain animal health [50]. *Shimia sp.*, another genus identified in healthy state in this study, has been reported to produce beneficial metabolites and degrade toxins [14]. Despite the fact that *Vibrio* was found in both states supporting previous studies [12, 22], it is considered floras opportunistic bacteria and cause disease when animal is under stress. Overall, the healthy genera captured in our study are comparable with earlier studies, confirming the correctness of our approach.

We also found several notable biomarkers in the disease state, including *Alteromonas*, *Photobacterium*, *Marinomonas* and *Pseudoalteromonas* which have previously been reported to cause WFD [1, 2, 26, 59]. Interestingly, these WFD related biomarkers are also associated with AHPND [8], a disease included in this study. In sum, the disease taxonomic biomarkers identified in this study correlate with previous studies, indicating their potential value in diagnosing disease onset.

Identifying the core microbes in the healthy and disease state reveals the essential microbes for multiple facets of microbiome-associated host functions. Several meta-analysis studies identified the core microbiome based on species presence in at least 10% of the samples [41]; however, we used a sample prevalence of 50% in our study. *Chryseomicrobium* and *Stenotrophomonas*, the healthy core genera identified in this study, are known for generating bioactive (anti-microbial and anti-enzyme) chemicals and, further, the glucosidase inhibitors produced by these genera give an additional advantage to survive in a competitive environment [42]. *Gemmobacter*, another core microbe identified in this study, was also reported to be present in the healthy conditions, though the functional status of this organism is not discussed [27]. Overall, the core genera catalogued in this study are associated with specific functional roles contributing to the growth of *P. vannamei*.

Microbes naturally coexist as a community rather than as individuals [10]. Hence, dysbiosis in the healthy consortia can favor the growth of opportunistic pathogens. Despite this, we lack a comprehensive understanding of the microbial communities that coexist and cooperate to help prevent bacterial infections. In our study, we cataloged the healthy and disease taxonomic biomarker, which frequently co-occurs and is critical for developing shrimp's particular facets. The knowledge of co-existing healthy genera catalogued in this study [Additional file 1: Table S9a] might be potentially applied for the management of aquaculture environment towards sustainable disease control.

Despite the advantages of amplicon-based sequencing, which includes microbial profiling and biomarker determination, it does not highlight the metabolic interactions [20]. With the vast number of genome sequences available, a whole genome-based approach was imperative to explore the metabolic interactions between healthy and disease states. This enabled the possibility of integrating genome-based metabolic modelling with an amplicon sequencing approach. Such genome-based approach provide insights into the taxonomic and functional interactions among the microbial communities [45, 53]. Hence, we further extended and enhanced our understanding of the identified taxonomic biomarkers by simulating the growth of biomarker species in diverse nutrient environments. Simulation through FBA allows the identification of healthy taxonomic biomarkers, which limit the growth of disease biomarkers. The constraint-based approach revealed five species: *Acinetobacter* sp WCHA55, *Acinetobacter tandoii* SE63, *Bifidobacterium pseudolongum* 49 D 6, *Brevundimonas pondensis* LVF1, and *Lutibacter profundi* LP1 limited the growth of the pathogen in a higher number of environments. The healthy biomarker species shortlisted with the metabolic modelling approach help steer the proliferation of pathogenic microorganisms and thereby control disease progression. The species from the genus *Acinetobacter* have been previously reported as a potential probiotic and believed to be safe for human health and could help replace antibiotics by controlling the pathogenic microorganisms and improve water quality in aquaculture ponds [18]. Similarly, the genera *Bifidobacteria* is another widely used probiotic in humans [23] and have also been reported to be present in healthy shrimp. Another shortlisted species, *Brevundimonas pondensis,* appears in various habitats, including aquatic environments [19], and has also been reported to be used for water pollutant treatment [34]. It should be interesting to observe the role of *Lutibacter profundi* LP1, which limited the growth of several disease-specific taxonomic markers used in our study. Belonging to the family *Flavobacteriaceae*, the members of this family are widely used in food and dairy products and are also associated with the degradation of organic matter in marine, seawater, and freshwater [52]. In summary, genome-scale simulation identified potential and novel candidate species that can be utilized as supplements in *P. vannamei* farming.

Analyzing the flux variability of pairwise communities revealed the role of *Acinetobacter* sp. in acetate production. The ability of *Acinetobacter* to produce acetate in all pairwise communities might explain its potential role as a health indicator. It is well established that organic acids are used in food preservation, as feed additives, and to control pathogens [39]. Although many beneficial or probiotic species are used in aquaculture, the lack of consistency and performance under different conditions is a major concern. Since microbial species interactions vary in different nutrient environments, it is crucial to capture the favorable environment that controls disease species' growth. As a result, our findings add value by demonstrating that sucrose facilitates parasitic interactions (i.e., limits disease growth), which is consistent with previous research that highlighted the role of sucrose and beneficial species in improved water quality, *P. vannamei* growth, and microbial composition.

## Conclusion

For the first time, 16S amplicon sequence data and genome-based metabolic modelling were combined for aquaculture application to find native biomarkers that may be best utilized to build probiotic formulations that leverage beneficial microorganisms for *P. vannamei* farming while limiting pathogen growth. Amplicon sequence-based analysis combined with metabolic modeling provided insights regarding metabolic interactions and the impact of nutrient environments, and finally, shortlist potential beneficial species to expedite experiments. We have employed this combination strategy for probiotic application in aquaculture, which was applied previously to human gut microbiota [25, 37, 60]. The key genera identified in this study could be used to prepare a formulation to replenish the healthy microbial consortia. In order to foster healthy shrimp farming, these essential genera can reduce the severity of disease conditions by removing opportunistic pathogenic bacteria and enhancing residential beneficial associations. Moreover, even though shotgun metagenomics can give a better taxonomic resolution and functional profile, we restricted our analysis to the amplicon dataset due to the limited availability of shotgun data with *P. vannamei*. Nevertheless, we can further enhance our understanding with curated genome scale metabolic models, followed by an experimental validation for the direct application of beneficial microbes in aquaculture farming.

## Supplementary Information

**Additional file 1.** **Table S1**. Initial Dataset collected for the study. **Table S2**. Final Dataset considered for the analysis. **Table S3**. List of healthy and disease assigned species obtained from BLASTP analysis and subjected to FBA. **Table S4**. Minimal media components used for building Genome scale metabolic models. **Table S5**. List of carbon sources used for simulation of models. **Table S6**. Alpha and Beta diversity indices for Individual datasets. **Table S7**. Alpha and Beta diversity indices for combined datasets. **Table S8**. List of Healthy and Disease biomarkers identified with LEfSe. **Table S9a**. List of Healthy Co-occurring genera identified with HiOrco. **Table S9b**. Co-ocurring genera identified with HiOrco in disease state. **Table S10**. List of short-listed communities with growth advantage of healthy species in at least five nutrient environments

**Additional file 2.** **Figure S1**. Comparison of Shannon diversity between healthy and disease state. The black line indicate the median value for each state. **Figure S2**. NMDS plot computed on the individual dataset based on Bray-Curtis distance for comparing microbial composition between healthy and disease dataset. **Figure S3**. NMDS plot on the combined dataset based on Bray Curtis to compare the microbial composition between healthy and disease state. **Figure S4**. Comparison of top 5 dominant phyla present in healthy and disease state. **Figure S5**. Venn diagram showing the number of genus-level taxa shared, and unique among healthy and disease state at 50% sample prevalence. **Figure S6**. LEfSe analysis depicting genus level biomarkers with a LDA sore > 2 at P < 0.05. The disease biomarkers are depicted with a negative score (red) and a positive LDA score (green) for healthy biomarkers.
