# Supplementary material for: In silico prediction of potential indigenous microbial biomarkers in Penaeus vannamei identified through meta-analysis and genome-scale metabolic modelling
Source: Environ Microbiome. 2023 Jan 11;18:2. doi: 10.1186/s40793-022-00458-6 (PMC9835370; doi:10.1186/s40793-022-00458-6)
Supplement: Supplementary file 2 — Additional file 2. Figure S1. Comparison of Shannon diversity between healthy and disease state. The black line indicate the median value for each state. Figure S2. NMDS plot computed on the individual dataset based on Bray-Curtis distance for comparing microbial composition between healthy and disease dataset. Figure S3. NMDS plot on the combined dataset based on Bray Curtis to compare the microbial composition between healthy and disease state. Figure S4. Comparison of top 5 dominant phyla present in healthy and disease state. Figure S5. Venn diagram showing the number of genus-level taxa shared, and unique among healthy and disease state at 50% sample prevalence. Figure S6. LEfSe analysis depicting genus level biomarkers with a LDA sore > 2 at P < 0.05. The disease biomarkers are depicted with a negative score (red) and a positive LDA score (green) for healthy biomarkers. [file 40793_2022_458_MOESM2_ESM.docx]

***In silico* prediction of potential indigenous microbial biomarkers in *Penaeus vannamei* identified through meta-analysis and genome-scale metabolic modelling**

**Additional file 2: Figures**

**Figure S1**
Comparison of Shannon diversity between healthy and disease state. The black line indicate the median value for each state. 
**Figure S2** 
NMDS plot computed on the individual dataset based on Bray-Curtis distance for comparing microbial composition between healthy and disease dataset
**Figure S3**
NMDS plot on the combined dataset based on Bray Curtis to compare the microbial composition between healthy and disease state
**Figure S4**
Comparison of top 5 dominant phyla present in healthy and disease state
**Figure S5**
Venn diagram showing the number of genus-level taxa shared, and unique among healthy and disease state at 50% sample prevalence
**Figure S6** 
LEfSe analysis depicting genus level biomarkers with a LDA sore > 2 at P < 0.05. The disease biomarkers are depicted with a negative score (red) and a positive LDA score (green) for healthy biomarkers.

**Figure S1**

**Comparison of Shannon diversity between healthy and disease state. The black line indicate the median value for each state.**

**
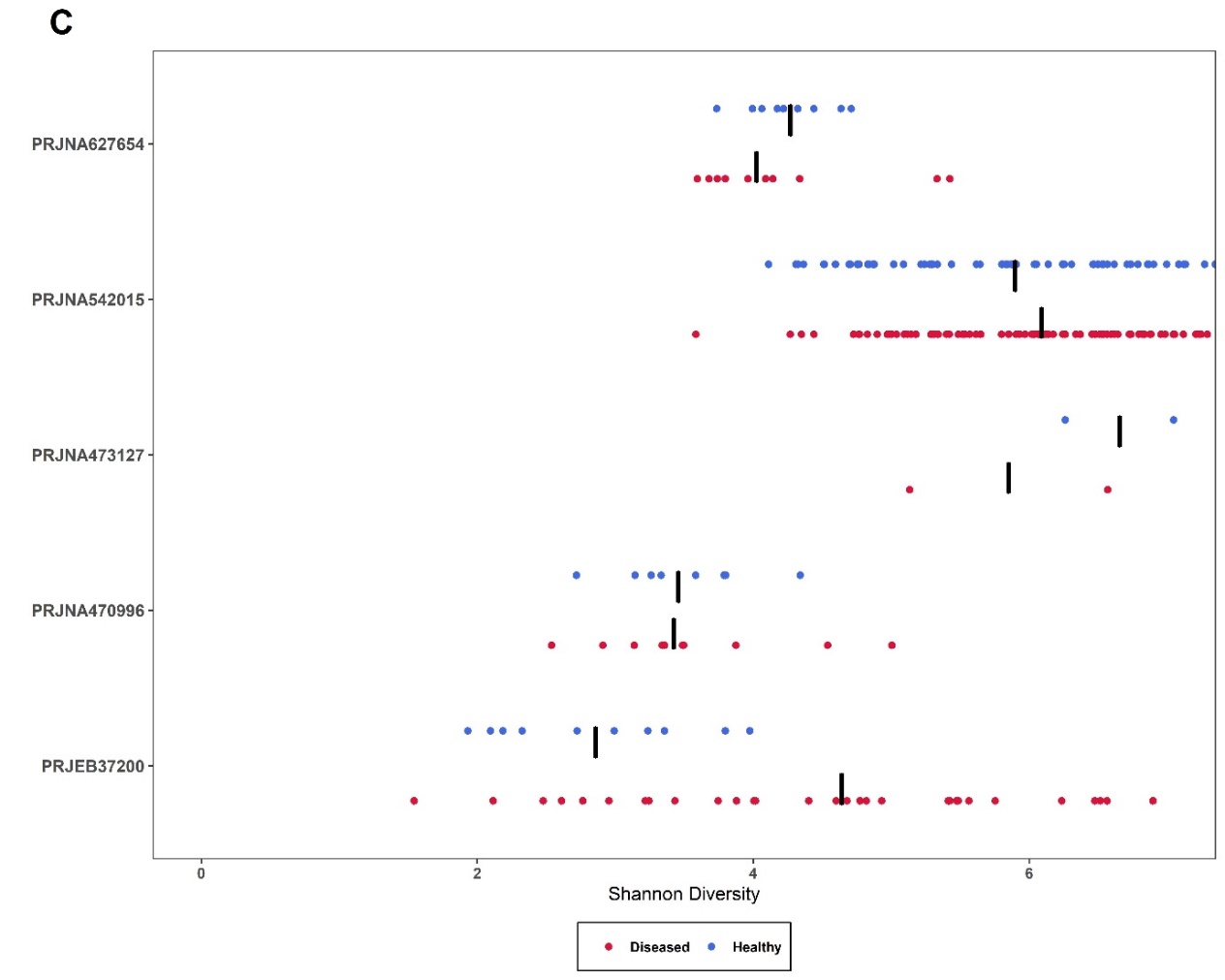
**

**Figure S2**

**NMDS plot computed on the individual dataset based on Bray-Curtis distance for comparing microbial composition between healthy and disease dataset**

**
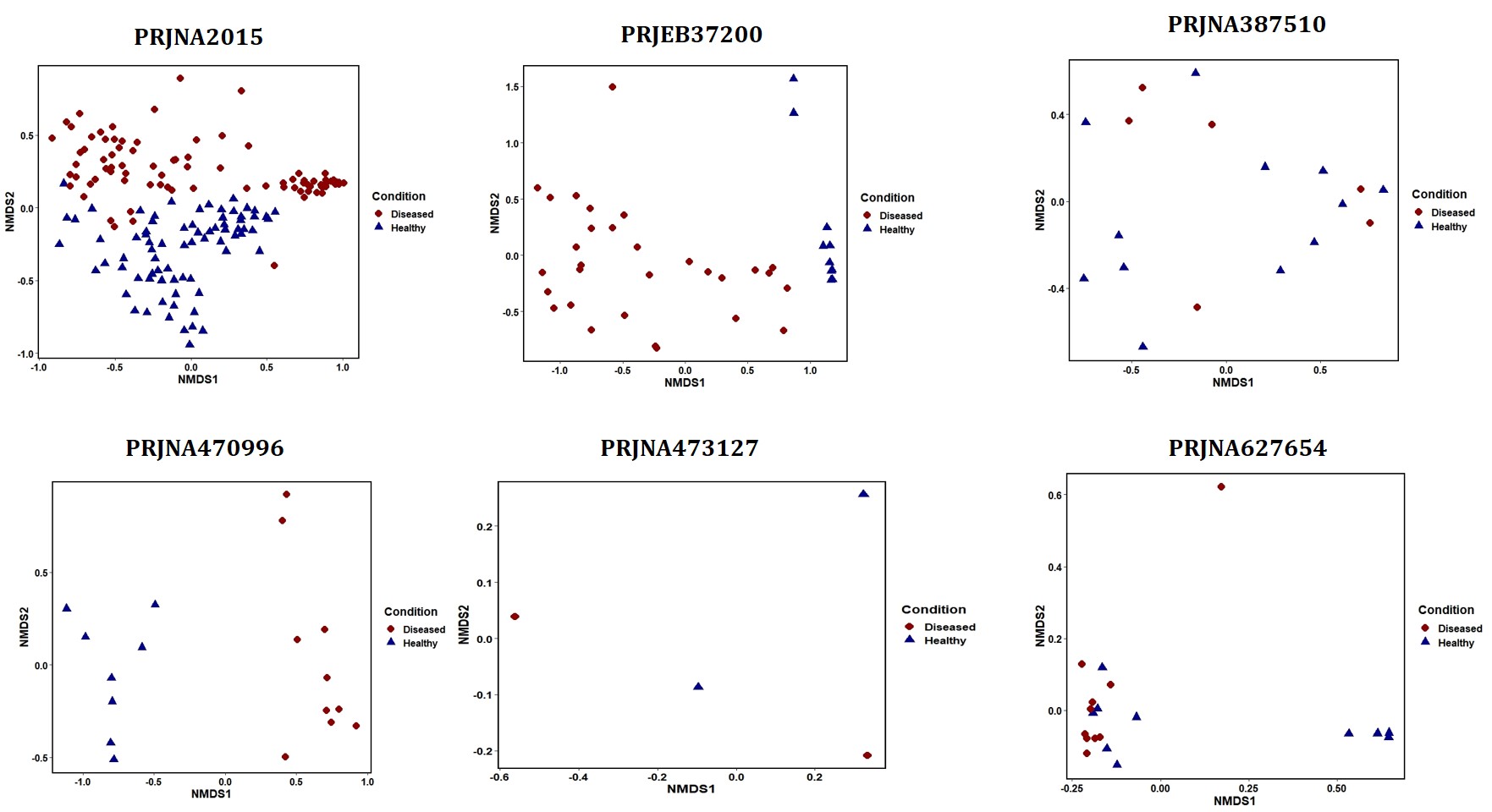
**

**Figure S3**

**NMDS plot on the combined dataset based on Bray Curtis to compare the microbial composition between healthy and disease state**

**
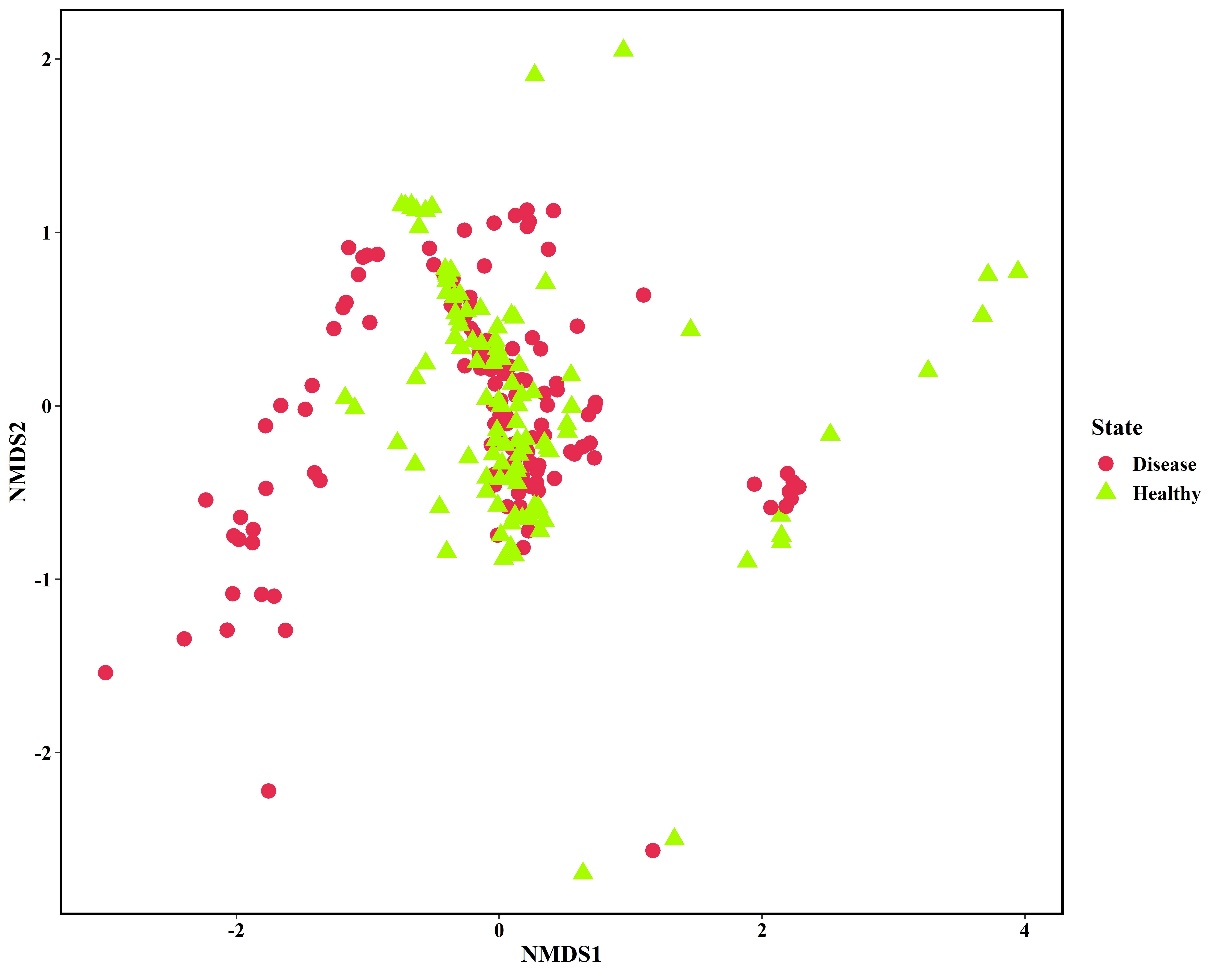
**

**Figure S4**

**Comparison of top 5 dominant phyla present in healthy and disease state**

**
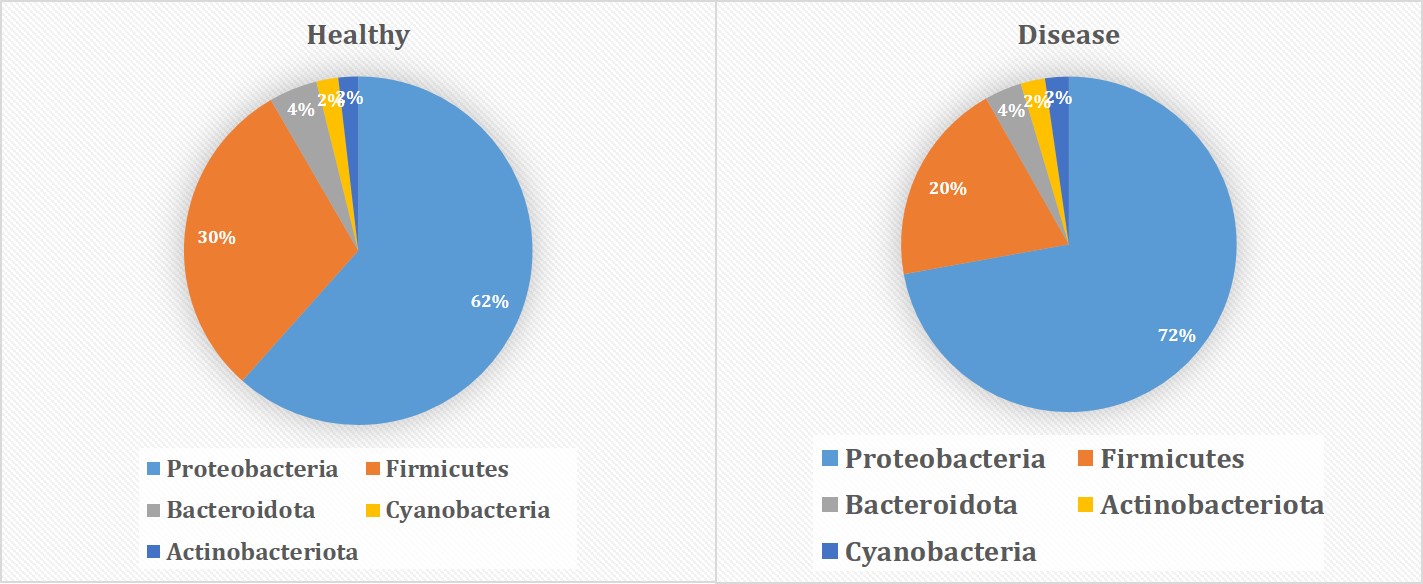
**

**Figure S5: Venn diagram showing the number of genus-level taxa shared, and unique among healthy and disease state at 50% sample prevalence**

**
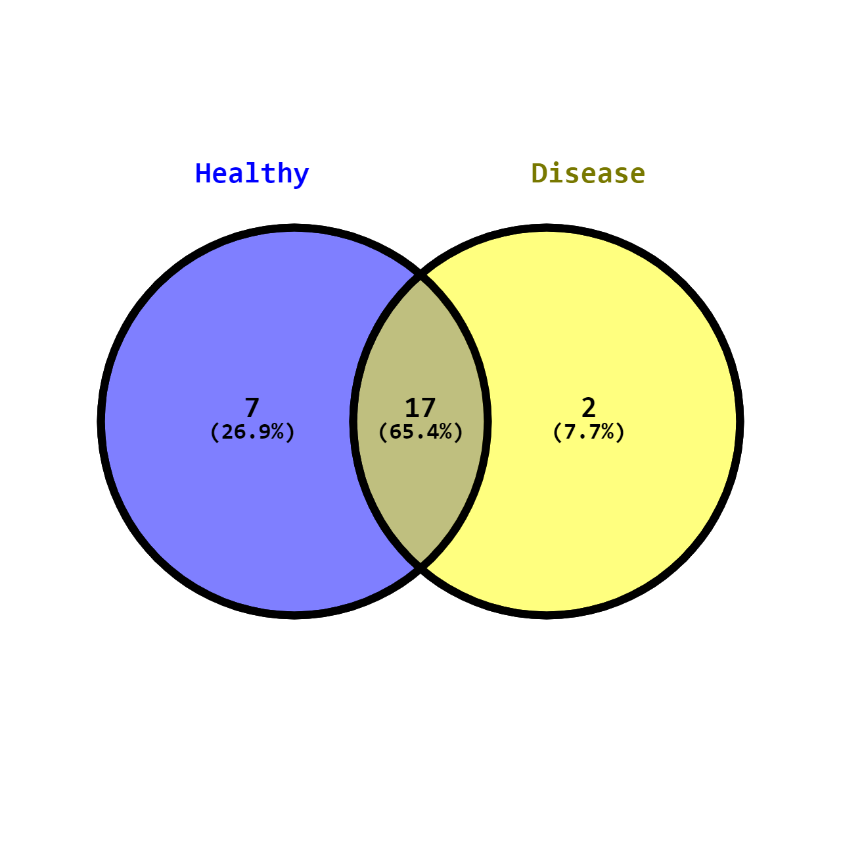
**

**Figure S6**

**LEfSe analysis depicting genus level biomarkers with a LDA sore > 2 at P < 0.05. The disease biomarkers are depicted with a negative score (red) and a positive LDA score (green) for healthy biomarkers.**

**
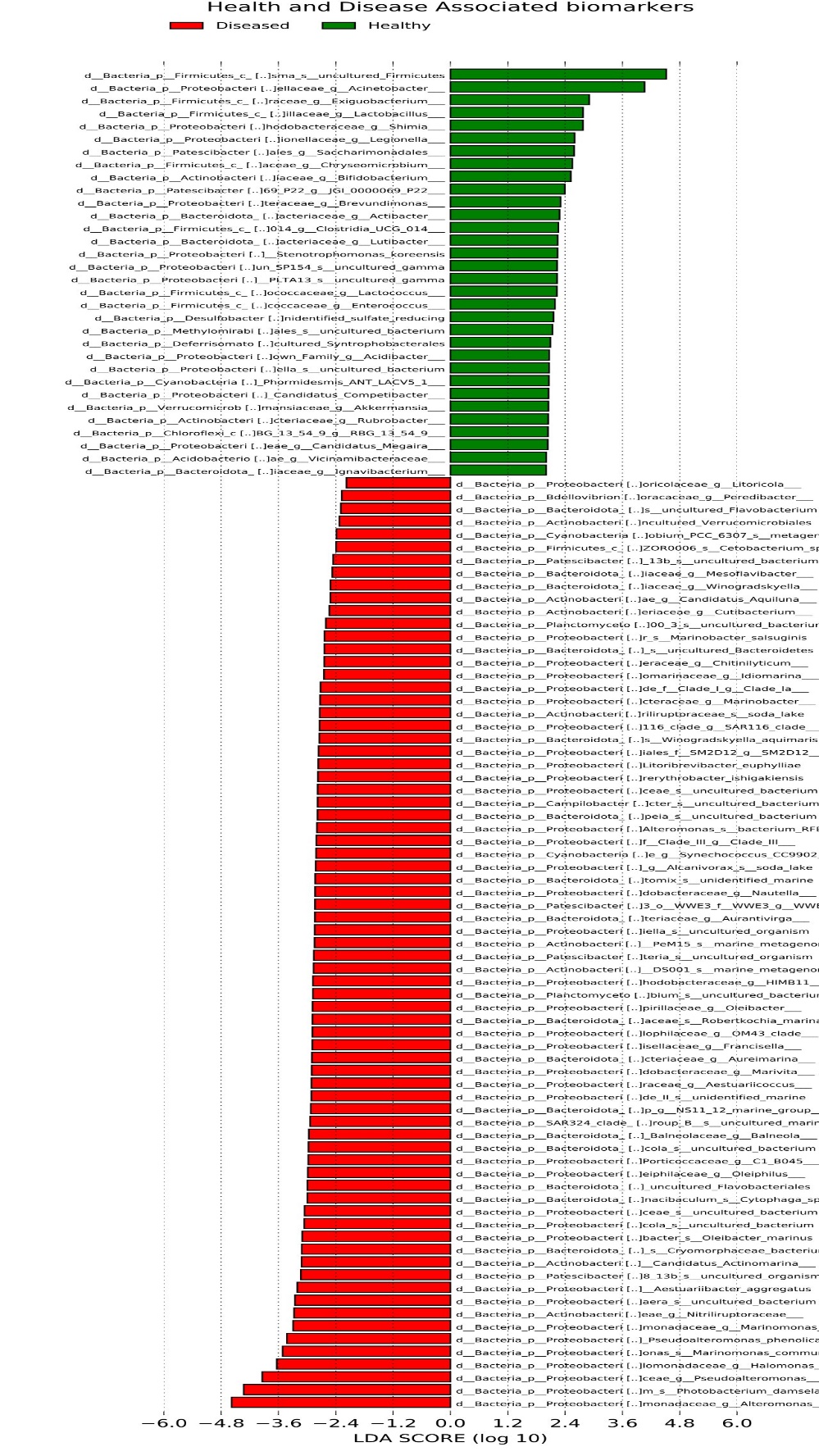
**
